# Impact of implementation intentions on physical activity practice in adults: A systematic review and meta-analysis of randomized clinical trials

**DOI:** 10.1371/journal.pone.0206294

**Published:** 2018-11-14

**Authors:** Marco Antonio Vieira da Silva, Thaís Moreira São-João, Valéria Cândido Brizon, Décio Henrique Franco, Fábio Luiz Mialhe

**Affiliations:** 1 Department of Community Dentistry, University of Campinas, Piracicaba Dental School, Piracicaba, São Paulo, Brazil; 2 School of Nursing, University of Campinas, Campinas, São Paulo, Brazil; Stanford University School of Medicine, UNITED STATES

## Abstract

**Objective:**

The aim of this study was to verify the efficacy of using theory-based strategies on implementation intentions in promoting physical activity (PA) among adults.

**Methods:**

This review was conducted in accordance with the PRISMA recommendations. The search was carried out in seven electronic databases (LILACS, PubMed, SciELO, Cochrane, Web of Science) and two searches of the “grey literature” were performed (Openthesis and OpenGrey). Randomized clinical trials (RCT), published up to September 2016, were considered eligible for this study. Two reviewers independently and systematically evaluated the eligibility criteria, and performed data extraction. A meta-analysis was performed for the purpose of comparing the effect between the intervention and control groups. The effect sizes were grouped in two subgroups with the purpose of more accurately verifying the effect caused by reinforcing the implementation intentions strategy, and using the inverse variance statistical method with random effects models to estimate the main effect of the implementation intention strategy on the PA behavior. Heterogeneity among the studies was evaluated by using I-square statistics, and the Jadad scale to evaluate the quality of included papers.

**Results:**

The search resulted in 12,147 records, of which 13 RCTs were considered eligible for this review. Sample age ranged from 18 to 76 years, and participants had conditions such as medullary lesion, coronary disease, obesity, diabetes mellitus, sedentarism or occupational stress. When the summary of the effect was analyzed in the meta-analysis, the result found in the subgroup with reinforcement of the implementation intentions strategy was 0.25 (IC 95% = 0.05–0.45) in favor of the intervention group. This demonstrated that application of the implementation intentions strategy was capable of increasing PA practice in the participants of these studies, in comparison with others that did not use this reinforcement.

**Conclusion:**

The findings of this review indicated that application of the theory of implementation intentions promoted PA behavior among the adults who received reinforcement of this strategy. The systematic review protocol was registered in the PROSPERO database under the number CRD42018090482.

## Introduction

Regular practice of physical activity (PA) promotes various benefits to health, among them increased cardiorespiratory and muscular fitness, reduced risk of coronary diseases, cancer, and improved control of diabetes mellitus [1–4]. Equally, reports have indicated an increase in health-related quality of life, contributing to improvement in daily activities at home and at work, in addition to favoring emotional well-being, thereby reducing depressive symptoms and increasing self-efficacy [5].

The World Health Organization (WHO) [2] has developed global PA recommendations, classifying adult individuals as active: when they practiced 150 minutes of aerobic PA of moderate intensity; or 75 minutes of intense PA; or the equivalent combination of activity of moderate and vigorous intensity. However, the importance of spreading this time over the course of the week must be taken into account; that is, some adaptive responses require daily regularity of PA [6]. This particularly applies to PA impact on insulin sensitivity; the maximum beneficial effect on this hormone occurs in a period of 12 to 48 hours after practice, and progressively returns to pre-activity levels in approximately three days [7]. The concentration of large volumes of PA in single sessions is known to be capable of triggering undesirable effects on health, such as lesions, wear and exhaustion [8].

In spite of the proven benefits of PA, a global study found that 31% of the world population is physically inactive [9]. In Brazil, the survey by Madeira et al. [10] revealed that 66.6% of the adults and 73.9% of older persons did not perform 150 minutes of PA per week [10]. The lack or low level of PA not only impacts on the health of persons, but promotes increased costs to the health systems all over the world [11].

Over the last few decades, with the purpose of contributing to the funding of PA, and other behaviors beneficial to health, diverse behavioral interventions, based on socio-cognitive theory models have been developed and evaluated as regards their efficacy and applicability in clinical practice [12]. Among these, the motivational models widely used in the area of health are outstanding, in which the intention (or motivation for performing a certain behavior) is understood as the main determinant of the action [13]. According to Ajzen [13], the philosopher responsible for creating the Theory of Planned Behavior (TPB), the intention is the main predictor of the behavior.

Although the intention is a strong predictor of physical activity behavior, there are reports of a hiatus or gap in the intention-behavior relationship; that is, even individuals with strong intentions about a certain objective fail when implementing them [14]. In a study conducted in patients with fibromyalgia, it was shown that even when they presented strong intention to practice PA, the motivation was only transformed into behavior in 32% of the participants [15].

Orbell and Sheeran [16] suggested that this “failure in behavior” could be explained by the fact that the stages of elaboration and executing a certain intention are, in truth, two distinct processes. Gollwitzer [17] argued that there are two stages involved in attaining a behavior: the motivational stage, in which a positive intention to perform a given behavior is elaborated, and a volitional stage, also named post-intention stage, in which the individual executes the intention already formed. This volitional stage was the basis for formulating the theory developed by Gollwitzer, named Implementation of the Intention, in which individuals formulate plans that specify ‘when, how and where’ they could begin to execute a given behavior. This theory presupposes that the more real the plans for performing a behavior, the greater the chances of it being effected [17].

From this perspective, the “action planning” strategy has been used with a view to operationalizing the theory of implementation intentions, which has been defined as a post-intention process, in which there is the need to plan when, how and where the behavior must be implemented. Action planning may be understood as a strategy that links responses (behavior) to anticipated situational pathways for performing them. Probably, persons who formulate this this planning have greater chances of acting in the intended manner, and also of more rapidly starting the desired behavior [18–19].

With the purpose of reinforcing the action planning and increasing the efficacy of performing the behavior, the use of anticipated mental stimulation of potential obstacles (or barriers) and ways of overcoming them has also been proposed; this process is named coping planning [20].

The aim of this study was to conduct a systematic review with meta-analysis of randomized clinical trials, in order to verify the efficacy of using theory-based strategies on implementation intentions in promoting PA among adults. Up to now, there is only one systematic review with meta-analysis in the literature, which addressed the same research topic [21]. In the cited article, the results demonstrated that the strategy of implementation intentions was able to promote the behavior of PA. However, an updated review that evaluates the effectiveness of this strategy is necessary, taking in account the most recent studies, as well as analyzing the design of the methodological approach among the studies that contributed to the performance of PA, particularly in relation to the application of reinforcements during the period of follow-up of the interventions and strategies for coping with obstacles.

## Materials and methods

To obtain the data, a systematic review of the literature with meta-analysis was performed. For writing the manuscript, the guidelines for Preferred Reporting Items for Systematic Reviews and Meta-Analyses (PRISMA) [22] and Cochrane were used [23]. The systematic review protocol was registered in the PROSPERO database under the number CRD42018090482.

### Study design and eligibility criteria

The study was a systematic review with the purpose of answering the following guiding question (based on the PICO strategy: “Would the strategy of implementation intentions (Intervention) be capable of promoting greater adherence to physical activity practice (Outcome) in adult individuals (Population) when compared with individuals who did not receive this strategy (Comparison)?”

RCTs published up to September 2016 were included, which included individuals over the age of 18 years and that applied the strategy of implementation intentions for the promotion of the practice of PA. All studies that applied the implementation intentions strategy, regardless of whether other interventions had been incorporated, were included. Due to scarcity of articles on implementation intentions reporting outcomes of interventions, no criteria for length of follow up were used. Reports published in English, Portuguese and Spanish language were included.

Studies excluded from the review were those that did not relate implementation intentions to the practice of PA; studies with individuals under the age of 18 years; observational studies; descriptive studies; non-controlled trials, editorial letters, pilot studies, historical commentaries, narrative, systematic and integrative reviews.

### Sources of information and search

The databases used for tracing the articles were PubMed (including MedLine), Cochrane Plus Library, Web of Science, Latin American and Caribbean Health Sciences (LILACS) and SciELO. The Openthesis and OpenGrey were used to capture “grey literature”, thereby avoiding selection bias.

To identify studies included in or considered for this review, a search strategy was developed for the above-mentioned electronic databases, using key words from a list of Descriptors in Health Sciences (DeCS) and in Medical Subject Headings (MeSH), and their combinations in the three languages—English, Portuguese and Spanish. For this purpose, the Boolean Operators AND and OR were used as follows: Implementação da intenção; Intention of implementing; La intención de implementar; Implementation intentions; Ativação da intenção; Intention activation; Activación de Intención; Técnicas de Planejamento; Planning Techniques; Técnicas de Planificación; Técnicas de implementação; Implementation Techniques; Técnicas de aplicación; Physical activity; Motor Activity; Actividad Motora; Atividade Motora; Atividade física. In order to assist and ensure that the search strategies were being carried out and finalized according to the PRISMA guideline, a librarian was consulted.

### Selection of studies

The selection of articles used in the study was performed in two stages: (1) abstracts and titles were selected and (2) the complete texts of the selected titles were obtained and read to determine the set of the final sample. The initial surveys were performed by two independent researchers who localized and selected the articles.

Initially, the researchers read the title of the article, and if this provided sufficient data for considering the article, it was included for reading in full (Stage 2), or excluded from the review, and its exclusion was justified. If the title of the article did not provide sufficient information for including or excluding it, the researchers read the abstract and opt between selecting or excluding the article by the abstract, and justify its exclusion. In Stage 2, the preliminarily eligible studies (by means of the title or abstract) had their text read in full, and evaluated with the purpose of verifying whether they fulfilled all the eligibility criteria. When these two reviewers did not reach agreement, a third reviewer was consulted for taking a final decision. If there were repetition of one and the same study, its copy would be excluded. The lists of references of the eligible articles were also checked independently by the two researchers to identify studies with potential relevance, which had not been found in the electronic search.

### Data collection and extraction process

After reading in full, the data of the eligible studies were extracted by two independent authors, by means of spreadsheets especially designed for data extraction. These included the following information: authors, year of publication, country, age, total sample, sex of participants, instrument used for applying the implementation intention strategy / frequency of reinforcement, instrument for measuring the physical activity behavior, risk/disease factor or lesion of participants, time of follow-up of the intervention, and evaluation of the quality of the article. Any disagreement was discussed, and a third review was consulted whenever necessary.

### Individual quality evaluation of the studies

The quality of the articles selected was evaluated independently by the two researchers, by means of the Jadad et al. scale [24] based on five parameters: 1.a—Was the study described as random (use of words such as "random", "chance", randomization")?; 1.b—Was the method adequate?; 2.a—Was the study described as double-blind?; 2.b—Was the method adequate? 3.—Was there a description of losses and exclusions? The score was attributed in the following manner: each item received one point for the response “yes”, or zero point for the response “no”. An additional point was attributed if, in item 1, the method for generating the randomization sequence was described and was adequate; in item 2, if the method of double-blind concealment was described and was adequate. One point was deducted if, in Question 1, the method for generating the randomization sequence was described, but in an inadequate manner; in Question 2, if the study was described as double-blind, but in an inadequate manner. The quality was evaluated according to the result based on the five parameters of the scale, thus, if the final score varied from 0 to 5 points, the stronger would be the study, and the better would be the methodological description. Therefore, the study would be considered strong when it received a score higher than or equal to 3.

### Outcome measures and data analysis

After tabulating the data, a meta-analysis was performed for the purpose of comparing the effect between the intervention and control groups. The effect sizes were grouped into two subgroups, using the inverse variance statistical method with random effects models [25] to estimate the main effect of the implementation intention strategy on the PA behavior. The united effect sizes were reported as difference of the standardized mean with their respective confidence intervals (CI) 95% and presented by means of figures represented in a graph named forest plot, which estimated the combined effect or magnitude of global effect represented by a diamond, to distinguish it from the isolated studies [26]. With the purpose of more accurately verifying the effect caused by reinforcing the implementation intentions strategy during the follow-up of the intervention, it was decided to perform a sensitivity analysis by means of forming two subgroups (a subgroup with studies that used reinforcement of the strategy and another subgroup that did not use it). Due to this subdivision of studies, the publication bias was not evaluated, as there were not enough studies to be grouped into a funnel plot.

When the information about means, standard deviation or exact number of participants was not described in the articles, they were acquired by means of contact with the corresponding author. The heterogeneity among the studies was calculated by means of I-square statistics (I^2^), in which the result is presented in percentage, which is the variable attributable to the heterogeneity among the studies [26]. The I² values were between 0% and 100%. The 0% values indicated absence of heterogeneity, while higher values demonstrated increase in heterogeneity. Although it is not an absolute rule, there is a suggestion for interpretation of the results: I² between 25–50% as low level of heterogeneity, between 50–75% as moderate and over 75% as high level of heterogeneity [27]. All the above-mentioned analyses were performed using the meta package implemented in the program R 3.4.4 for Windows [28].

## Results

### Literature search

During the first stage of selecting the studies, 532 records were found, distributed among the five electronic databases. After removing the repeated/duplicated articles, 507 remained for analysis of the titles and abstracts. Eleven thousand six hundred and fifteen (11.615) studies from the “grey literature” were found by the Openthesis and OpenGrey search strategies, however, only eight were related to aim of this study. After analyzing the titles and abstract, only 39 studies were eligible for analysis of the complete text. However, of the 39 studies read in full, only 13 were included in this systematic review, which complied with the recommendation that of the literature to be included there must be at least 30% of the articles that fulfilled the inclusion criteria established [29]. For the meta-analysis, 11 articles were selected. The references of the studies initially eligible were carefully evaluated to verify the possibility of an article absent from the main search strategy; however, no article was included after checking the references. The search strategy for PubMed database is presented in S1 Appendix. PRISMA Checklist is presented in S1 Table.

**Fig 1:**
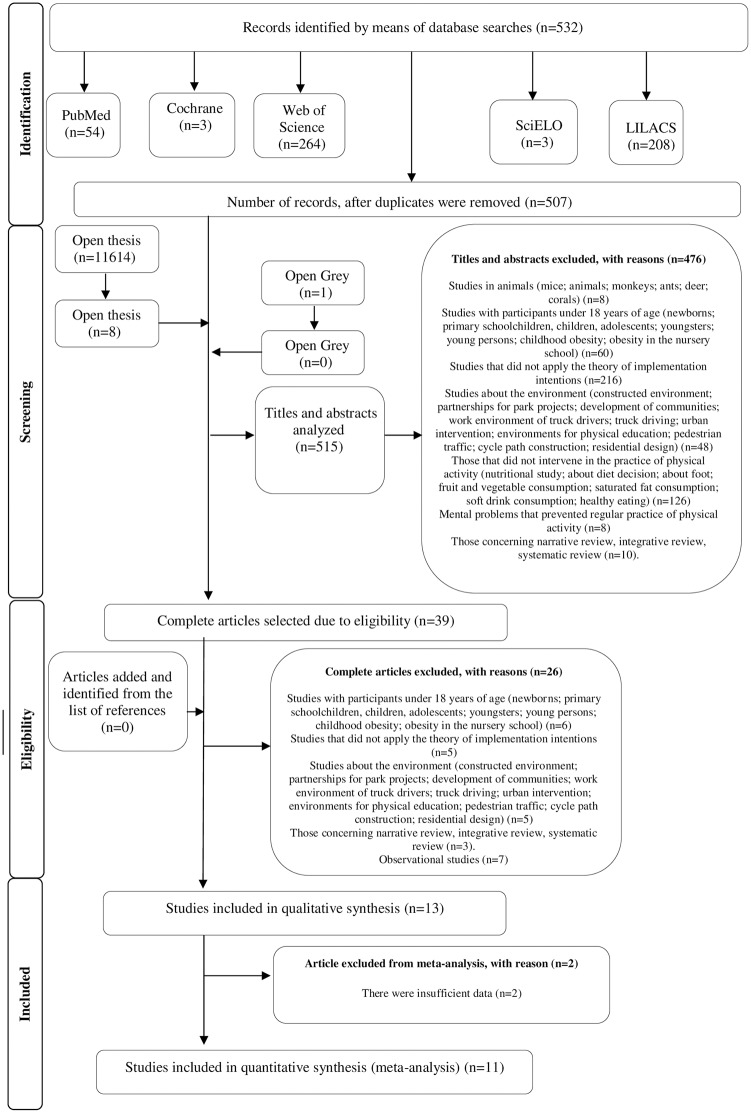
Flow diagram of the selection of the studies.

Fig 1 reproduces the process of search, identification, inclusion and exclusion of the articles.

Among the articles included, the authors observed a diversity of researchers in different countries (two from Canada, two from the United States, one from Holland, one from Germany, two from Poland, three from the United Kingdom and two from Brazil) which worked with the topic proposed in this review, in spite of all the studies found being described in the English language. In addition to the country of origin, all the studies selected were categorized with regard to authors, year of publication, age, sample size; sex of participants; instruments used for application of the strategy of implementation intentions; frequency and mode of reinforcement; instrument for measurement of PA behavior, risk factor, disease or lesion of participants; time of follow-up in weeks, and evaluation of quality by the Jadad et al scale 24 (Table 1).

**Table 1:** Characteristics of studies selected for this review.

AUTHOR/YEAR	AGE/Years	SEX	CONDITION OF HEALTH	COUNTRY	SAMPLE SIZE	STRATEGIES USED FOR IMPLEMENTATION INTENTIONS	FREQUENCY AND MODE OF REINFORCEMENT	INSTRUMENT FOR MEASUREMENT OF PHYSICAL ACTIVITY	TIME OF FOLLOW-UP (in weeks)	QUALITY ASSESSMENT—Jadad
Arbour and Ginis 2004 [30]	mean: 46.6 years	Female	Sedentarism	Canada	47	Action planning	No reinforcement was applied	Godin-Shephard Leisure-Time Physical Activity Questionnaire	8	3
Budden and Sagarin 2007 [31]	18 to 74 years	Both	Occupational stress	United States of America	635	Action planning	No reinforcement was applied	Self-report of behavior	Not reported	3
De Vet et al. 2009 [32]	18 to 65 years (mean: 45.9)	Both	Obesity	Holland	709	Action planning	Face-to-face reinforcement after 2 and 12 weeks since baseline	Dutch Short Questionnaire to Assess Health enhancing physical activity (SQUASH)	24	3
Godin et al. 2010 [33]	18 to 55 years (mean: 37.6)	Both	Obesity	Canada	349	Action planning	No reinforcement was applied	Leisure-time physical activity	12	3
Latimer et al. 2006 [34]	18 to 65 years (mean: 41.1)	Both	Medullary lesion	United States of America	54	Action planning and coping planning	Telephone reinforcement after 4 weeks since baseline	Physical Activity Recall Assessment for Individuals with SCI (PARA–SCI)	8	3
Luszczynska et al. 2007 [35]	18 to 76 years (mean: 44.01)	Female	Obesity	Poland	55	Action planning and coping planning	No reinforcement was applied	Self-report of behavior	8	3
Luszczynska et al. 2006 [36]	18 to 67 years (mean: 54)	Both	Coronary diseases	Poland	114	Action planning	Telephone reinforcement after 8 weeks since baseline	Self-report of behavior	32	3
Milne et al. 2002 [37]	18 to 34 years (mean: 20.04)	Both	no health problem	United Kingdom	248	Action planning	No reinforcement was applied	Self-report of behavior	2	2
Prestwich et al. 2009 [38]	mean: 23.76 years	Both	Sedentarism	United Kingdom	155	Action planning	Reinforcement via text message by mobile phone (did not specify time)	Self-report of behavior	4	3
Prestwich et al. 2010 [39]	mean: 23.44 years	Both	no health problem	United Kingdom	149	Action planning	Reinforcement via text message by mobile phone, during the first three consecutive weeks	Self-report of behavior	4	3
Rodrigues et al. 2013 [40]	Mean Control Group: 61.8 years (mean: Intervention Group 56.7 years	Both	Coronary diseases	Brazil	136	Action planning and coping planning	Face-to-face reinforcement after 4 weeks and telephone reinforcement after 15 e 45 days since baseline	Habitual Physical Activity Questionnaire (Baecke)	8	3
Silva et al. 2015 [4]	Mean Control Group: 59.87 years (mean: Intervention Group 61.27 years	Both	Diabetes mellitus	Brazil	30	Action planning and coping planning	Telephone reinforcement after 4 weeks since baseline	International Physical Activity Questionnaire (IPAQ)	8	3
Stadler et al. 2009 [41]	30 to 50 years (mean: 41.28)	Female	no health problem	Germany	256	Action planning and coping planning	No reinforcement was applied	Self-report of behavior	16	3

### Study characteristics

In the final sample, thirteen studies were evaluated. The mean age of the study participants ranged between 20.0 and 61.8 years. As regards sex, only three studies [30,35,41] worked exclusively with women, the others worked with men and women.

The majority of the studies (92%) reported a very heterogeneous intervention follow-up period, ranging from a minimum of two to a maximum of thirty-two weeks. As regards the sample size, there was also a high level of heterogeneity, ranging between 30 to 709 participants, in which 77% intervened in specific target populations related to some type of disease or lesion (medullary lesion [34], coronary diseases [36,40], obesity [32–33,35] and diabetes mellitus [4]) or risk factor (sedentarism [30,38] and occupational stress [31]) and 23% intervened in healthy adult populations [37,39,41].

Relative to the type of instrument used for measuring PA practice, 100% of the studies used self-report instruments to assess behavior. Among these, 46% applied self-report instruments validated in the literature [4,30,32–34,40]; that is, questionnaires that presented adequate measurement properties in previous studies (reproducibility and validity), such as the Habitual PA questionnaire of Baecke (QAFH-Baecke) [40], the International Physical Activity Questionnaire (IPAQ) [4], the Godin-Shephard Leisure-Time Physical Activity Questionnaire [30], the Dutch Short Questionnaire to Assess Health Enhancing Physical Activity (SQUASH) [32], the Physical Activity Recall Assessment for Individuals with SCI (PARA–SCI) [34] and the Leisure-time physical activity [33]. However, over half (54%) of the studies [31,35–39,41] applied non-validated self-report instruments, in which they asked the participants to respond about the duration and/or frequency of PA.

As regards the type of strategy used to put into practice the theory of implementation intentions, the participants prepared plans for the purpose of effectuating the intended behavior (action planning), as well as anticipated mental plans for coping with possible obstacles that could impede the behavior in question (coping planning). Thus, 61% of the studies [30–33,36–39] applied action planning only and 39% [4,34–35,40–41] associated action planning with coping planning. Between baseline and final follow-up 53% of the studies [4,32,34,36,38–40], received reinforcement of the initial plans. These reinforcements were applied both by presence [32,40], and via telephone contact [4,34,36,40] and text messages via mobile phones [38–39].

With respect to the professionals who applied the interventions at baseline and at reinforcement times, only two studies [40,41] mentioned adequate training for this conduct. Relative to the qualification of the professionals who applied the strategies, in one of the studies the interventionist was a nurse [40] and in the other, a psychologist plus a specialist in cardiac rehabilitation [36]. Both studies [36,40] dealt with participants who had coronary disease.

As regards the control group, the majority of the studies (92%) applied a motivational phase to their participants through videos [4,30], pamphlets [34,37,38,41], face-to-face meetings [32,35,36,40], postal mail [33], or text messages via phone cell [39]. This purpose of this motivational phase was to inform participants about the importance of PA, and its different modalities, frequency and duration.

### Methodological analysis of studies

For the analysis of methodological quality of the studies, the authors used the checklist proposed by Jadad et al. [24], with a score ranging from 0 to 5. Of the thirteen articles, twelve presented Score 3, considered very good, showing that the methodology of the articles was well described. Only one article [37] obtained Score 2, because it did not report whether or not the study was double-blind and did not report losses and exclusions.

### Meta-analysis of the effect of interventions on the practice of physical activity

**Fig 2:**
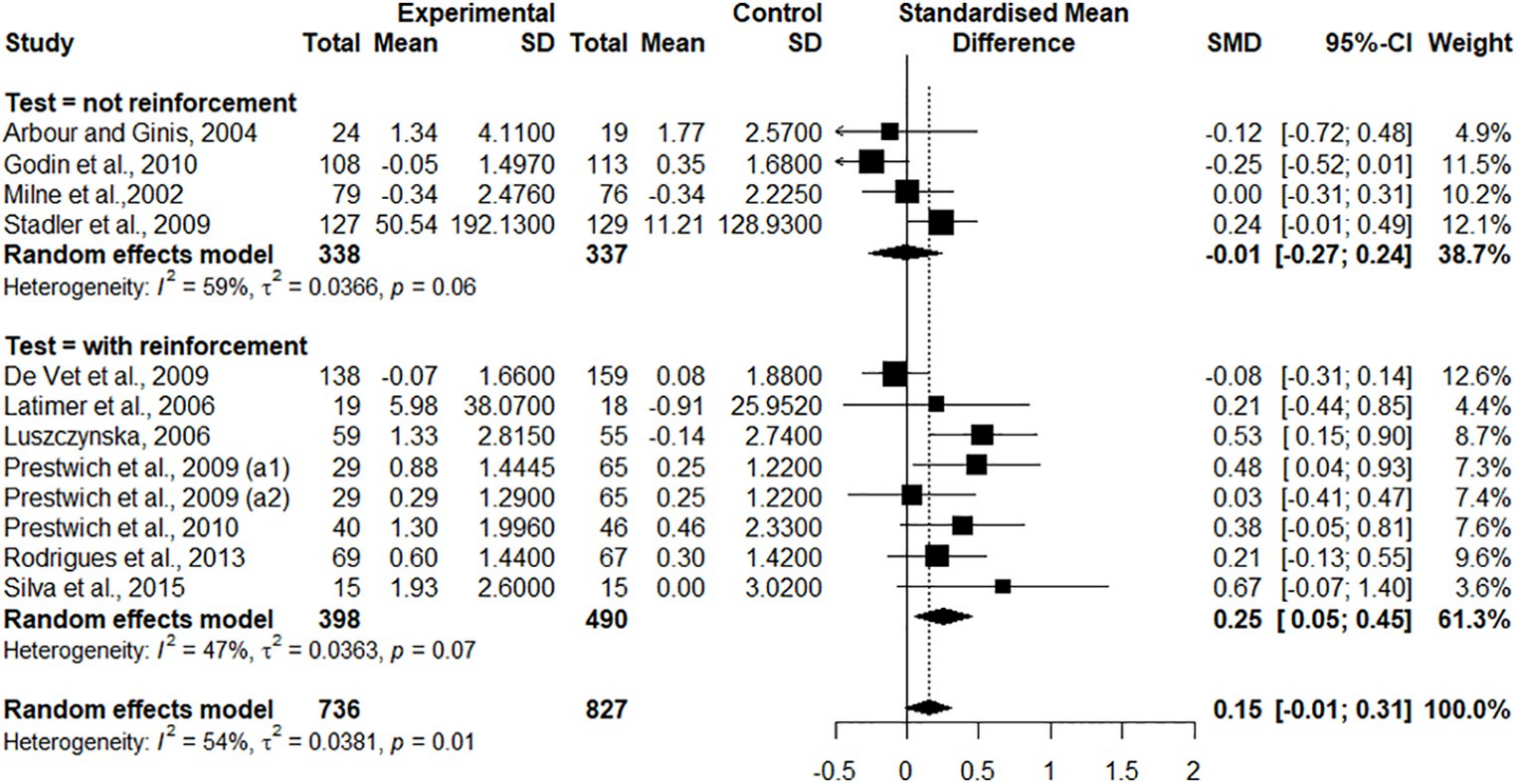
Forest plot of studies submitted to meta-analysis. (a1 and a2) = with reference to the study of Prestwich et al. [38].

For the meta-analysis, eleven randomized studies were used [4,30,32–34,36–41]. Only two articles [31,35] did not enter into the analysis, because there were insufficient data and there was no response to the email sent to the authors for clarification. In the study of Prestwich et al. [38], two results in the analysis (a1 and a2) were used, because there was the presence of two control groups (pure control and another associated with a motivational study based on the Theory of Motivational Protection–PMT-) and two intervention groups (one that worked with the strategy of implementation intentions and the other with the implementation intentions associated with text messages). Thus, due to the subdivision in the study of Prestwich et al. [38] into two analyses, twelve results were totaled in the meta-analysis (Fig 2).

The effect size of the studies presented variation that ranged between -0.25 and 0.67 demonstrating that the heterogeneity was moderate (I^2^ = 54%) [27]. In the general analysis, when the summary of the effect was analyzed, the result shown was 0.15 (IC 95% = -0.01–0.31) demonstrating that the implementation intentions strategy did not significantly increase the practice of PA in comparison with the groups that did not receive this strategy. However, when each subgroup of the present meta-analysis (groups in studies that received reinforcement of implementation intentions strategy, and groups in studies that did not received it) was analyzed separately, in the groups that received reinforcement of the strategy, the result shown was 0.25 (IC 95% = 0.05–0.45) demonstrating that application of reinforcement of the implementation intentions strategy was capable of increasing PA practice in a statistically significant way in comparison with the groups that did not receive the reinforcement (Fig 2).

## Discussion

In view of the need for in-depth assessment and up-dating of the topic about interventions that used the theory of implementation intentions, this study aimed to conduct a systematic review of randomized clinical trials with meta-analysis to verify the efficacy of the strategy implementation intentions on PA promotion among adults. Major findings suggested that the strategy was effective in promoting PA behavior in studies with individuals who received reinforcement of the implementation intentions strategy in periods of different lengths of time, ranging from 2 to 34 weeks; with heterogeneous sample sizes, of both sexes, and specific diseases. All studies used self-report assessments, such as questionnaires, to measure health behaviors.

The implementation intentions model was composed of two strategies: action planning and coping planning [17]. Action planning aimed to raise awareness in the individual as regards possible future situations in which the behavior could be achieved and the possible responses to these situations, making explicit when, where, and how the individual would perform the behavior. Coping planning focused on risky situations or barriers that might impede, interfere with, or complicate the achievement of the target behavior and ways of overcoming these barriers [20].

With regard to the planning prepared for increasing adherence to PA behavior, 39% of the studies [4,34–35,40–41] associated action planning with coping planning. Coping planning is an important mechanism to enable the individual to establish effective responses in the face of problems that show up [20]. In the five studies of the present review that used this type of planning, the mean age of participants was over 40 years. According to Scholz et al. [20] application of the strategy of implementation intentions, by means of preparing plans for coping with obstacles may be more effective in older individuals, because they have more experience of life in comparison with younger persons. Therefore, they are able to overcome the barriers more easily, because they have had more time and maturity to develop the skills necessary for dealing with adversities.

With respect to follow-up of the intervention, 53% of the studies [4,32,34,36,38–40] received reinforcement of the plans during the course of the research, and these reinforcements were significant for promotion of PA practice, since the interventions that used them were responsible for over half of the total weight of the meta-analysis (61.3%). It was also shown that among the reinforcements applied, those that resulted in greater weight were those provided by means of telephone contact [4,34,36,40]. Further to the use of reinforcements, the study of Latimer et al. [34] proposed that in these reinforcements, the plans should be updated, so that if necessary, there would be flexible adjustments in response to the eventual changes in the participants’ lifestyles. Corroborating this finding, the study of Budden and Sagarin [31] emphasized that they did not obtain significant changes in the PA behavior in the experimental group, due to inflexibility of the plans executed.

In relation to the quality of this review, of the thirteen articles included in the final sample, only one study [37] presented the final value of below 3 on the Jadad et al. scale [24], thus showing the inclusion of good quality investigations, with adequate description of sample selection methodology, blinding, development of the studies and presentation of their results, as has been recommended by the guidelines [22].

In dealing with the moderate heterogeneity found in the result of the meta-analysis (I^2^ = 54%), this may possibly be attributed to the difference relative to the period of follow-up of the interventions (from two to thirty-two weeks), and it may also have been caused by the different characteristics of health of the populations included in each study (medullary lesions, coronary diseases, diabetes mellitus, sedentary, occupational stress, obesity and healthy adults), thereby resulting in moderate variability among the studies.

With regard to the four studies [30–33] that presented no significant differences in the group that received the strategy of implementation intentions, the absence of the use of coping planning, was factor in common among them, thus demonstrating the importance of obstacle management until the behavior has indeed been transformed into habit among the participants. Moreover, with regard to the application of strategies based on behavioral theories the mere effect of the measure must be taken into consideration. This concerns a phenomenon also named the question-behavior effect, which refers to the impact of asking questions about a behavior (versus not asking these questions) on the subsequent performance of this behavior; that is, the simple fact of being asked about the behavior makes subjects reflect on the issue and change their attitude or practice. Frequently, the absence of significant difference between intervention vs. control groups, and the implementation of behavior among the controls has been attributed to this effect [42]. Another factor that may have led to no significant difference in these studies may have been the fact that in the majority of them (92%), the control groups received a motivational phase regarding the importance of the practice of PA, and thus, may have contributed to individuals adopting this behavior without having participated in the implementation intentions strategy.

As a limitation to the results shown in the present review, the measurement of PA by means of self-report instruments must be pointed out, whether or not they have been validated in previous studies. Thus, the variability in the methodological strictness used to measure the mentioned behavior could have affected the power of detecting significant differences among the studies, thus corroborating the results of the systematic review of Bélanger-Gravel et al. [21]. Moreover, self-report instruments, due to their subjective nature, present considerable limitations, such as memory bias and social desirability, making it necessary to ally them to objective instruments such as accelerometers, pedometers, movement sensors, among others, to precisely measure the levels of PA. In this sense, social desirability is understood as the tendency of respondents to distort self-reports in a favorable direction, and this might compromise self-reports on PA, by overestimating them [43]. Corroborating this finding, 54% of the studies of the present review [4,32–33,36,38,40–41] reported the absence of objective measurements as the main limitation of the research. Apart from the above-mentioned limitation, two others were pointed out by the studies more frequently: interventions programs with short follow-up times [4,30,40], and reduced numbers of participants [4,30], possibly harming the external validity and applicability of the studies. The fact that most of the studies did not consider whether the interventionist who applied the implementation intentions strategy had received training for this conduct, and whether they were qualified for it, may be considered a limitation for their applicability in future research.

## Conclusion

The results of this review with meta-analysis suggested that the application of the strategy of implementation intentions seemed to promote PA behavior in different populations and countries, particularly when there was incorporation of plans for management of perceived obstacles. The use of planning reinforcement during follow-up of the intervention also appeared to be effective both for engaging in and maintaining PA practice.

## Supporting information

S1 Appendix Search strategy for PubMed database used in this research. (DOCX)

S1 Table PRISMA checklist. (DOC)
