# Supplementary material for: Impact of implementation intentions on physical activity practice in adults: A systematic review and meta-analysis of randomized clinical trials
Source: PLoS One. 2018 Nov 14;13(11):e0206294. doi: 10.1371/journal.pone.0206294 (PMC6235272; doi:10.1371/journal.pone.0206294)
Supplement: S1 Appendix — (DOCX) [file pone.0206294.s001.docx]

**Supporting Information – PubMed: Full Search Strategy**

For purposes of reproducibility, the search strategy for PubMed database is described below. In order to conduct these specific searches, the standardized descriptors of Medical Subject Headings (MeSH) were used; i.e., – (((("Planning Techniques"[Mesh] OR Planning Technique OR Technique, Planning OR Techniques, Planning OR Planning Technic OR Planning Technics OR Technic, Planning OR Technics, Planning OR Methodology, Planning OR Methodologies, Planning OR Planning Methodologies OR Planning Methodology OR Planning Theories OR Planning Theory OR Theories, Planning OR Theory, Planning)) AND ("Motor Activity"[Mesh] OR Activities, Motor OR Activity, Motor OR Motor Activities OR Physical Activity OR Activities, Physical OR Activity, Physical OR Physical Activities OR Locomotor Activity OR Activities, Locomotor OR Activity, Locomotor OR Locomotor Activities))) AND ((((Intention of implementing) OR Implementation intentions) OR Intention activation) OR Implementation Techniques))). In this database, 54 articles were found.
