# Supplementary material for: Impact of implementation intentions on physical activity practice in adults: A systematic review and meta-analysis of randomized clinical trials
Source: PLoS One. 2018 Nov 14;13(11):e0206294. doi: 10.1371/journal.pone.0206294 (PMC6235272; doi:10.1371/journal.pone.0206294)
Supplement: S1 Table — (DOC) [file pone.0206294.s002.doc]

| **Section/topic** | **#** | **Checklist item** | **Reported on page #** |
| --- | --- | --- | --- |
| **TITLE** | | |  |
| Title | 1 | Identify the report as a systematic review, meta-analysis, or both.  **“Impact of implementation intentions on physical activity practice in adults: a systematic review and meta-analysis of randomized clinical trials”** | 1 |
| **ABSTRACT** | | |  |
| Structured summary | 2 | Provide a structured summary including, as applicable: background; objectives; data sources; study eligibility criteria, participants, and interventions; study appraisal and synthesis methods; results; limitations; conclusions and implications of key findings; systematic review registration number.  **We have used the PLOS abstract format as requested by the PLOS author guidelines.** | 2-3 |
| **INTRODUCTION** | | |  |
| Rationale | 3 | Describe the rationale for the review in the context of what is already known.  **“Up to now, there is only one systematic review with meta-analysis in the literature, which addressed the same research topic [21]. In the cited article, the results demonstrated that the strategy of implementation intentions was able to promote the behavior of PA. However, an updated review that evaluates the effectiveness of this strategy is necessary, taking in account the most recent studies, as well as analyzing the design of the methodological approach among the studies that contributed to the performance of PA, particularly in relation to the application of reinforcements during the period of follow-up of the interventions and strategies for coping with obstacles.”** | 5 |
| Objectives | 4 | Provide an explicit statement of questions being addressed with reference to participants, interventions, comparisons, outcomes, and study design (PICOS).  **“The study was a systematic review with the purpose of answering the following guiding question (based on the PICO strategy: “Would the strategy of implementation intentions (Intervention) be capable of promoting greater adherence to physical activity practice (Outcome) in adult individuals (Population) when compared with individuals who did not receive this strategy (Comparison)?”**  **RCTs published up to September 2016 were included, which included individuals over the age of 18 years and that applied the strategy of implementation intentions for the promotion of the practice of PA.”** | 6 |
| **METHODS** | | |  |
| Protocol and registration | 5 | Indicate if a review protocol exists, if and where it can be accessed (e.g., Web address), and, if available, provide registration information including registration number.  **“The study was registered at the International Prospective Register of Systematic Reviews (PROSPERO) with number** **CRD42018090482.”** | 3 |
| Eligibility criteria | 6 | Specify study characteristics (e.g., PICOS, length of follow-up) and report characteristics (e.g., years considered, language, publication status) used as criteria for eligibility, giving rationale.  **“The study was a systematic review with the purpose of answering the following guiding question (based on the PICO strategy: “Would the strategy of implementation intentions (Intervention) be capable of promoting greater adherence to physical activity practice (Outcome) in adult individuals (Population) when compared with individuals who did not receive this strategy (Comparison)?”**  **RCTs published up to September 2016 were included, which included individuals over the age of 18 years and that applied the strategy of implementation intentions for the promotion of the practice of PA. All studies that applied the implementation intentions strategy, regardless of whether other interventions had been incorporated, were included. Due to scarcity of articles on implementation intentions reporting outcomes of interventions, no criteria for length of follow up were used. Reports published in English, Portuguese and Spanish language were included.**  **Studies excluded from the review were those that did not relate implementation intentions to the practice of PA; studies with individuals under the age of 18 years; observational studies; descriptive studies; non-controlled trials, editorial letters, pilot studies, historical commentaries, narrative, systematic and integrative reviews.”** | 6 |
| Information sources | 7 | Describe all information sources (e.g., databases with dates of coverage, contact with study authors to identify additional studies) in the search and date last searched.  **“The databases used for tracing the articles were PubMed (including MedLine), Cochrane Plus Library, Web of Science, Latin American and Caribbean Health Sciences (LILACS) and SciELO. The Openthesis and OpenGrey were used to capture “grey literature”, thereby avoiding selection bias.** **RCTs published up to September 2016 were included.”** | 6 |
| Search | 8 | Present full electronic search strategy for at least one database, including any limits used, such that it could be repeated.  **The search strategy for PubMed database is presented in S1 Appendix.** | 10 |
| Study selection | 9 | State the process for selecting studies (i.e., screening, eligibility, included in systematic review, and, if applicable, included in the meta-analysis).  **“The selection of articles used in the study was performed in two stages: (1) abstracts and titles were selected and (2) the complete texts of the selected titles were obtained and read to determine the set of the final sample. The initial surveys were performed by two independent researchers who localized and selected the articles.**  **Initially, the researchers read the title of the article, and if this provided sufficient data for considering the article, it was included for reading in full (Stage 2), or excluded from the review, and its exclusion was justified. If the title of the article did not provide sufficient information for including or excluding it, the researchers read the abstract and opt between selecting or excluding the article by the abstract, and justify its exclusion. In Stage 2, the preliminarily eligible studies (by means of the title or abstract) had their text read in full, and evaluated with the purpose of verifying whether they fulfilled all the eligibility criteria. When these two reviewers did not reach agreement, a third reviewer was consulted for taking a final decision. If there were repetition of one and the same study, its copy would be excluded. The lists of references of the eligible articles were also checked independently by the two researchers to identify studies with potential relevance, which had not been found in the electronic search.”** | 7-8, Fig 1 |
| Data collection process | 10 | Describe method of data extraction from reports (e.g., piloted forms, independently, in duplicate) and any processes for obtaining and confirming data from investigators.  **“After reading in full, the data of the eligible studies were extracted by two independent authors, by means of spreadsheets especially designed for data extraction. These included the following information: authors, year of publication, country, age, total sample, sex of participants, instrument used for applying the implementation intention strategy / frequency of reinforcement, instrument for measuring the physical activity behavior, risk/disease factor or lesion of participants, time of follow-up of the intervention, and evaluation of the quality of the article. Any disagreement was discussed, and a third review was consulted whenever necessary.”** | 8 |
| Data items | 11 | List and define all variables for which data were sought (e.g., PICOS, funding sources) and any assumptions and simplifications made.  **See table 1.** | 12-13 |
| Risk of bias in individual studies | 12 | Describe methods used for assessing risk of bias of individual studies (including specification of whether this was done at the study or outcome level), and how this information is to be used in any data synthesis.  **“The quality of the articles selected was evaluated independently by the two researchers, by means of the Jadad et al. scale [24] based on five parameters: 1.a - Was the study described as random (use of words such as "random", "chance", randomization")?; 1.b - Was the method adequate?; 2.a - Was the study described as double-blind?; 2.b - Was the method adequate? 3. - Was there a description of losses and exclusions? The score was attributed in the following manner: each item received one point for the response “yes”, or zero point for the response “no”. An additional point was attributed if, in item 1, the method for generating the randomization sequence was described and was adequate; in item 2, if the method of double-blind concealment was described and was adequate. One point was deducted if, in Question 1, the method for generating the randomization sequence was described, but in an inadequate manner; in Question 2, if the study was described as double-blind, but in an inadequate manner. The quality was evaluated according to the result based on the five parameters of the scale, thus, if the final score varied from 0 to 5 points, the stronger would be the study, and the better would be the methodological description. Therefore, the study would be considered strong when it received a score higher than or equal to 3.”** | 8-9 |
| Summary measures | 13 | State the principal summary measures (e.g., risk ratio, difference in means).  **“After tabulating the data, a meta-analysis was performed for the purpose of comparing the effect between the intervention and control groups. The effect sizes were grouped into two subgroups, using the inverse variance statistical method with random effects models [25] to estimate the main effect of the implementation intention strategy on the PA behavior.”** | 9 |
| Synthesis of results | 14 | Describe the methods of handling data and combining results of studies, if done, including measures of consistency (e.g., I^2^) for each meta-analysis.  **“After tabulating the data, a meta-analysis was performed for the purpose of comparing the effect between the intervention and control groups. The effect sizes were grouped into two subgroups, using the inverse variance statistical method with random effects models [25] to estimate the main effect of the implementation intention strategy on the PA behavior. The united effect sizes were reported as difference of the standardized mean with their respective confidence intervals (CI) 95% and presented by means of figures represented in a graph named forest plot, which estimated the combined effect or magnitude of global effect represented by a diamond, to distinguish it from the isolated studies [26]. With the purpose of more accurately verifying the effect caused by reinforcing the implementation intentions strategy during the follow-up of the intervention, it was decided to perform a sensitivity analysis by means of forming two subgroups (a subgroup with studies that used reinforcement of the strategy and another subgroup that did not use it). Due to this subdivision of studies, the publication bias was not evaluated, as there were not enough studies to be grouped into a funnel plot.**  **When the information about means, standard deviation or exact number of participants was not described in the articles, they were acquired by means of contact with the corresponding author. The heterogeneity among the studies was calculated by means of I-square statistics (I2), in which the result is presented in percentage, which is the variable attributable to the heterogeneity among the studies [26].** **All the above-mentioned analyses were performed using the meta package implemented in the program R 3.4.4 for Windows [28].”** | 9-10 |

Page 1 of 2

| **Section/topic** | **#** | **Checklist item** | **Reported on page #** |
| --- | --- | --- | --- |
| Risk of bias across studies | 15 | Specify any assessment of risk of bias that may affect the cumulative evidence (e.g., publication bias, selective reporting within studies).  **“Due to this subdivision of studies, the publication bias was not evaluated, as there were not enough studies to be grouped into a funnel plot.”** | 9 |
| Additional analyses | 16 | Describe methods of additional analyses (e.g., sensitivity or subgroup analyses, meta-regression), if done, indicating which were pre-specified.  **“With the purpose of more accurately verifying the effect caused by reinforcing the implementation intentions strategy during the follow-up of the intervention, it was decided to perform a sensitivity analysis by means of forming two subgroups (a subgroup with studies that used reinforcement of the strategy and another subgroup that did not use it).”** | 9 |
| **RESULTS** | | |  |
| Study selection | 17 | Give numbers of studies screened, assessed for eligibility, and included in the review, with reasons for exclusions at each stage, ideally with a flow diagram.  **“During the first stage of selecting the studies, 532 records were found, distributed among the five electronic databases. After removing the repeated/duplicated articles, 507 remained for analysis of the titles and abstracts. Eleven thousand six hundred and fifteen (11.615) studies from the “grey literature” were found by the Openthesis and OpenGrey search strategies, however, only eight were related to aim of this study. After analyzing the titles and abstract, only 39 studies were eligible for analysis of the complete text. However, of the 39 studies read in full, only 13 were included in this systematic review, which complied with the recommendation that of the literature to be included there must be at least 30% of the articles that fulfilled the inclusion criteria established [29]. For the meta-analysis, 11 articles were selected. The references of the studies initially eligible were carefully evaluated to verify the possibility of an article absent from the main search strategy; however, no article was included after checking the references.”** | 10, Fig 1 |
| Study characteristics | 18 | For each study, present characteristics for which data were extracted (e.g., study size, PICOS, follow-up period) and provide the citations.  **This information is summarized in the text and extensively presented in table 1** | 12-13 |
| Risk of bias within studies | 19 | Present data on risk of bias of each study and, if available, any outcome level assessment (see item 12).  **“For the analysis of methodological quality of the studies, the authors used the checklist proposed by Jadad et al. [24], with a score ranging from 0 to 5. Of the thirteen articles, twelve presented Score 3, considered very good, showing that the methodology of the articles was well described. Only one article [37] obtained Score 2, because it did not report whether or not the study was double-blind and did not report losses and exclusions.”** | 15,  Table 1 |
| Results of individual studies | 20 | For all outcomes considered (benefits or harms), present, for each study: (a) simple summary data for each intervention group (b) effect estimates and confidence intervals, ideally with a forest plot.  **“The effect size of the studies presented variation that ranged between -0.25 and 0.67 demonstrating that the heterogeneity was moderate (I2=54%) [27]. In the general analysis, when the summary of the effect was analyzed, the result shown was 0.15 (IC 95% = -0.01-0.31) demonstrating that the implementation intentions strategy did not significantly increase the practice of PA in comparison with the groups that did not receive this strategy.** **However, when each subgroup of the present meta-analysis (groups in studies that received reinforcement of implementation intentions strategy, and groups in studies that did not received it) was analyzed separately, in the groups that received reinforcement of the strategy, the result shown was 0.25 (IC 95% = 0.05-0.45) demonstrating that application of reinforcement of the implementation intentions strategy was capable of increasing PA practice in a statistically significant way in comparison with the groups that did not receive the reinforcement.”** | 16-17, Fig 2 |
| Synthesis of results | 21 | Present results of each meta-analysis done, including confidence intervals and measures of consistency.  **“The effect size of the studies presented variation that ranged between -0.25 and 0.67 demonstrating that the heterogeneity was moderate (I2=54%) [27]. In the general analysis, when the summary of the effect was analyzed, the result shown was 0.15 (IC 95% = -0.01-0.31) demonstrating that the implementation intentions strategy did not significantly increase the practice of PA in comparison with the groups that did not receive this strategy. However, when each subgroup of the present meta-analysis (groups in studies that received reinforcement of implementation intentions strategy, and groups in studies that did not received it) was analyzed separately, in the groups that received reinforcement of the strategy, the result shown was 0.25 (IC 95% = 0.05-0.45) demonstrating that application of reinforcement of the implementation intentions strategy was capable of increasing PA practice in a statistically significant way in comparison with the groups that did not receive the reinforcement.”** | 16-17, Fig 2 |
| Risk of bias across studies | 22 | Present results of any assessment of risk of bias across studies (see Item 15).  **“Due to this subdivision of studies, the publication bias was not evaluated, as there were not enough studies to be grouped into a funnel plot.”** | 9 |
| Additional analysis | 23 | Give results of additional analyses, if done (e.g., sensitivity or subgroup analyses, meta-regression [see Item 16]).  **“When each subgroup of the present meta-analysis (groups in studies that received reinforcement of implementation intentions strategy, and groups in studies that did not received it) was analyzed separately, in the groups that received reinforcement of the strategy, the result shown was 0.25 (IC 95% = 0.05-0.45) demonstrating that application of reinforcement of the implementation intentions strategy was capable of increasing PA practice in a statistically significant way in comparison with the groups that did not receive the reinforcement.”** | 16-17, Fig 2 |
| **DISCUSSION** | | |  |
| Summary of evidence | 24 | Summarize the main findings including the strength of evidence for each main outcome; consider their relevance to key groups (e.g., healthcare providers, users, and policy makers).  “**Major findings suggested that the strategy was effective in promoting PA behavior in studies with individuals who received reinforcement of the implementation intentions strategy in periods of different lengths of time, ranging from 2 to 34 weeks; with heterogeneous sample sizes, of both sexes, and specific diseases. All studies used self-report assessments, such as questionnaires, to measure health behaviors.”** | 17 |
| Limitations | 25 | Discuss limitations at study and outcome level (e.g., risk of bias), and at review-level (e.g., incomplete retrieval of identified research, reporting bias).  **Limitations are discussed in detail on pages 19 and 20.** | 19-20 |
| Conclusions | 26 | Provide a general interpretation of the results in the context of other evidence, and implications for future research.  **“The results of this review with meta-analysis suggested that the application of the strategy of implementation intentions seemed to promote PA behavior in different populations and countries, particularly when there was incorporation of plans for management of perceived obstacles. The use of planning reinforcement during follow-up of the intervention also appeared to be effective both for engaging in and maintaining PA practice.”** | 20 |
| **FUNDING** | | |  |
| Funding | 27 | Describe sources of funding for the systematic review and other support (e.g., supply of data); role of funders for the systematic review.  **In accordance to the PLOS instructions to authors, we do not report Funding in the manuscript (“Do not include funding sources in the Acknowledgments or anywhere else in the manuscript file. Funding information should only be entered in the financial disclosure section of the online submission system.”)** | NR |
